# Effects on the Motor Function, Proprioception, Balance, and Gait Ability of the End-Effector Robot-Assisted Gait Training for Spinal Cord Injury Patients

**DOI:** 10.3390/brainsci11101281

**Published:** 2021-09-28

**Authors:** Ji Cheol Shin, Ha Ra Jeon, Dahn Kim, Sung Il Cho, Won Kyu Min, June Sung Lee, Da Som Oh, Jeehyun Yoo

**Affiliations:** 1 Department and Research Institute of Rehabilitation Medicine, Severance Hospital, Yonsei University College of Medicine, Yonsei-ro 50-1, Seodaemun-gu, Seoul 03722, Korea; jcsevrm@yuhs.ac (J.C.S.); dahn.dana.kim@gmail.com (D.K.); 2 Department of Physical Medicine and Rehabilitation, National Health Insurance Service Ilsan Hospital, Ilsan-ro 100, Ilsandong-gu, Goyang-si 10444, Korea; jeon1021@nhimc.or.kr; 3 Rehabilitation Center, Inje University Ilsan Paik Hospital, Juhwa-ro 170, Ilsanseo-gu, Goyang-si 10380, Korea; jo@paik.ac.kr; 4 Department of Rehabilitation Center, Severance Hospital, Yonsei University College of Medicine, Yonsei-ro 50-1, Seodaemun-gu, Seoul 03722, Korea; MWK10003@yuhs.ac; 5 Department of Rehabilitation Center, National Health Insurance Service Ilsan Hospital, Ilsan-ro 100, Ilsandong-gu, Goyang-si 10444, Korea; asarabia@nhimc.or.kr; 6 Inje Industry, Academic Cooperation Foundation, Inje-ro 197, Gimhae-si 50834, Korea; dasom654@naver.com; 7 Department of Rehabilitation Medicine, Inje University Ilsan Paik Hospital, Juhwa-ro 170, Ilsanseo-gu, Goyang-si 10380, Korea; 8 Yonsei University College of Medicine, Yonsei-ro 50-1, Seodaemun-gu, Seoul 03722, Korea

**Keywords:** robotics, robot-assisted gait training, spinal cord injuries, proprioception, postural balance

## Abstract

The primary aim of this study was to reveal the effects of end-effector robot-assisted gait training (RAGT) on motor function, proprioception, balance, and gait ability in patients with incomplete spinal cord injury (SCI). The secondary aim was to determine the correlation between clinical outcomes. This study was a prospective and multi-center study. A total of 13 incomplete SCI patients who met inclusion criteria received 30 min of RAGT with Morning Walk^®^ (Curexo, Seoul, South Korea), and 1 h of conventional physiotherapy 5 times per week for 4 weeks. Clinical outcome measures were 10 m walk test (10MWT), 6 min walk test (6mWT), lower extremity motor score (LEMS), proprioception, Berg Balance Scale (BBS), and Walking Index for Spinal Cord Injury (WISCI)-II. All participants were assessed within 48 h before and after the intervention. All clinical outcomes were statistically improved after RAGT. Subgroup analysis according to the initial proprioception, WISCI-II in the normal group showed a statistically significant improvement compared to the abnormal group. Initial BBS and WISCI-II had a positive correlation with most of the final clinical outcomes. The final BBS had a strong positive correlation with the final 10MWT, 6mWT, and WISCI-II. Initial proprioception had a positive correlation with the final WISCI-II. The final proprioception also had a moderate positive correlation with 6mWT and BBS. This study’s results suggest that the end-effector RAGT could promote proprioception, balance ability and walking ability. Postural control ability and proprioception also had a positive relationship with gait ability.

## 1. Introduction

The spinal cord plays an important role in connecting the brain and peripheral nerves. It transfers an ascending sensory signal from the periphery to the brain via the ascending tract (spinothalamic tract, posterior column, and medial lemniscus pathway) or descending motor signals from the brain to the periphery via the descending tract (corticospinal tract). If the spinal cord is injured by a traumatic or non-traumatic cause, the patient can have various degrees of motor and/or sensory impairment below the level of injury.

For human gait, lower extremity muscle power, joint proprioception, visual balance, and cognition are needed. Balance ability can also be affected by joint proprioception and visual function. After spinal cord injury (SCI), patients experience various degrees of proprioceptive impairment. This is essential for locomotor recovery and skill learning after SCI [1,2,3,4,5,6].

Physicians should consider locomotor training for patients with incomplete SCI with some degree of motor function in their lower extremities. In the past, overground walking training with or without gait aid and/or physical assistance was the only intervention method. However, their therapeutic quality and duration are easily affected by the patients’ motor functions. It is difficult to facilitate repetitive and physiological gait patterns by physiotherapists’ physical assistance. Body-weight-supported treadmill training (BWSTT) is the next training method. A harness supports patients’ body weight and provides more support than a gait aid. However, BWSTT still requires physical assistance from a physiotherapist to make the swing phase or support the stance phase [7,8,9].

In the late 1990s, RAGT was introduced in clinical practice, which is an exoskeletal-type robot (Lokomat^®^) with a treadmill base. RAGT allows the patient to experience physiological gait patterns repetitively and safely with body weight support by a harness [9]. An end-effector type robot has also been developed and is used in the clinical field. Unlike the exoskeletal-type robot, which links the ankle, knee, and hip joints to the robot, the end-effector robot attaches only the feet to the footplate [10]. Therefore, it allows free movement of the knee and hip joints and provides “destabilization training.” This training can reinforce the neuronal circuit and contribute to postural control and sensory integration [11,12,13,14].

RAGT in patients with incomplete SCI revealed improvements in mobility-related outcomes (gait endurance and Walking Index for Spinal Cord Injury [WISCI]-II) and lower extremity motor strength compared to conventional physiotherapy [7,9,12]. However, recent RAGT studies for incomplete SCI patients have used Lokomat^®^, except for three articles [15,16,17,18].

The primary aim of this study was to reveal the effects of end-effector RAGT on motor function, proprioception, balance, and gait ability in patients with incomplete SCI. The secondary aim was to determine which initial clinical outcome correlates with the final outcome and the correlation between final outcomes in patients with SCI.

## 2. Materials and Methods

### 2.1. Participants

This study was a prospective, multi-center study. Three hospitals participated: Inje University Ilsan Paik Hospital and the National Health Insurance Service Ilsan Hospital, which are secondary hospitals, and Yonsei University Severance Hospital, a tertiary hospital. Between May 2020 and July 2021, all SCI patients admitted to the Department of Physical Medicine and Rehabilitation of the three hospitals were evaluated.

Inclusion criteria were: (1) traumatic or non-traumatic SCI over the age of 19 years, (2) onset of less than 6 months, (3) upper motor neuron injury with the neurological level of injury from C2 to T12, and (4) ASIA Impairment Scale (AIS) of C or D. Exclusion criteria included joint contracture of the lower extremity, fracture risk with severe osteoporosis, pressure injuries of the sacrum, ischium or coccyx, severe cognitive impairment, combined peripheral neuropathy, gait problem before the SCI, and WISCI-II level 20.

The study was approved by (1) the Inje University Ilsan Paik Hospital Institutional Review Board (IRB), No. 2020-06-006; (2) the National Insurance Service Ilsan Hospital IRB, NHIMC 2020-05-005-007; and (3) the Yonsei University Severance Hospital IRB, No.4-2020-0542. All participants were informed of the study purpose, intervention protocol, and outcome measures before they signed an informed consent form.

### 2.2. Intervention

**Figure 1:**
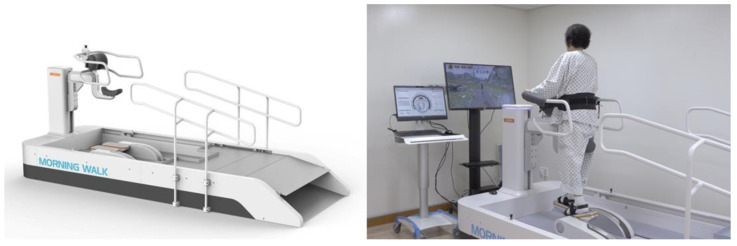
The Morning Walk^®^ (Curexo, Seoul, South Korea) which is an end-effector type robot. It uses a saddle for body-weight support.

Participants received RAGT with Morning Walk^®^ (Figure 1, Curexo, Seoul, South Korea). This is an end-effector type robot and the first gait training robot to use a saddle for weight support; previous robots used harnesses. Patients can access the robot in a wheelchair and move directly to the saddle. Therefore, it takes less time to get on and off the robot than previous gait training robots, which is the advantage of the Morning Walk^®^. It produces a gait cycle according to the trajectory of the footplate. Only the patient’s feet were attached to the robot, and the knee and hip joints moved freely. In front of the Morning Walk^®^, there is a virtual reality screen. During RAGT, patients can experience walking through a park or forest because it provides visual feedback according to the gait speed.

All participants received 30 min of RAGT and 1 h of conventional physiotherapy five times per week for 4 weeks (20 sessions in total). RAGT operates in the ground-level gait training mode. The participants started with a cadence of 30 steps/min, a step length of 30 cm, and 20% body weight support (BWS). These parameters were adjusted according to the individual’s performance during RAGT. Cadence was increased by 5 steps/min if a patient could perform RAGT for 10 min without resting. The step length was adjusted according to cadence. The BWS was determined based on the average BWS of the prior session. Conventional physiotherapy consisted of sitting and standing balance training, sit-to-stand training, and strengthening exercises.

### 2.3. Outcome Measures

We evaluated gait speed (10 m walk test, 10MWT) and endurance (6 min walk test, 6mWT). Participants who were unable to walk were considered 10MWT as 0 m/s and 6mWT as 0 m. In addition, we evaluated muscle strength (lower extremity motor score (LEMS) of the International Standards for Neurological Classification of Spinal Cord Injury (ISNCSCI]), proprioception (proprioception of the ISNCSCI at the ankle and knee), balance ability (Berg Balance Scale, BBS), and walking ability (Walking Index for Spinal Cord Injury-II (WISCI-II)). Proprioception evaluation of the ISNCSCI recommends using the great toe, ankle, and knee. According to the ISNCSCI, manual, proprioception (joint movement appreciation and position sense) is graded as absent, impaired, or normal. A grade of 0 (absent) indicates that the patient is unable to correctly report joint movement on large movement of the joint. A grade of 1 (impaired) indicates that the patient can consistently report joint movement with 8 of 10 correct answers only on large movement of the joint. A grade of 2 (normal) indicates that the patient can consistently report joint movement with 8 of 10 correct answers both small (approximately 10° of motion) and large movement [19]. Our purpose in evaluating proprioception was to reveal the effects of the end-effector robot on proprioception. The great toe has no movement during RAGT by the end-effector robot. Therefore, we decided to use only ankle and knee proprioception in the ISNCSCI for outcome measures. All participants were assessed within 48 h before and after the intervention.

### 2.4. Statistical Analysis

After checking whether the data followed a normal distribution by Shapiro-Wilk test, we used non-parametric tests. Comparisons of pre- and post-RAGT effects in all participants were assessed using the Wilcoxon signed-rank test. Subgroup analysis was according to the initial proprioception status, the Wilcoxon signed-rank test was performed for within-group comparisons of RAGT effects and the Mann-Whitney U test was performed for between-group comparisons. We calculated the effect size at alpha = 0.05 with 80% power by using the G*Power version 3.1.9.6 software (Heinrich Heine University Düsseldorf, Düsseldorf, Germany). Spearman’s correlation was used to determine the correlation between the clinical outcome measures. SPSS ver. 25.0 software (IBM SPSS Inc., Armonk, NY, USA) was used for statistical analyses. Statistical significance was considered when the *p*-value was less than 0.05.

## 3. Results

### 3.1. Demographic Data

A total of 13 patients, including eight males and five females, participated in this study. Their median age was 52 years (age range 19–85 years), and the median time from onset was 48 days (range 19–139 days). Twelve patients were injured by a traumatic cause and the other injured by a non-traumatic cause. Ten patients had tetraplegia with AIS D, one had paraplegia with AIS C, and one had tetraplegia with AIS D (Table 1). After 20 sessions of RAGT, paraplegia with AIS C improved to AIS D.

### 3.2. Outcome Measures after RAGT

After 20 sessions of RAGT, all outcome measures, including 10MWT, 6mWT, LEMS, proprioception of the ankle and knee, BBS, and WISCI-II, had significantly improved (*p* = 0.002, 0.002, 0.003, 0.027, 0.001, and 0.001, respectively; Table 2).

Before RAGT, only three participants could perform the 10MWT and 6mWT, and their WISCI-II level was 8 or 13. The other participants could not perform the 10MWT and 6mWT because of their low WISCI-II level (0–4). After RAGT, only one participant could not perform the 10 MWT or 6mWT. He had the lowest WISCI-II level after RAGT among the participants, which was 2.

Before RAGT, six participants had intact proprioception of the ankle and knee, and seven participants had decreased proprioception of the ankle or knee. As mentioned above, we hypothesized that the end-effector robot could assist proprioception and balance improvement by providing destabilization training. Therefore, we performed a subgroup analysis based on the initial proprioception status to compare the outcome measures.

### 3.3. Outcome Measures According to the Initial Proprioception Status

We divided the participants into two groups according to their initial proprioception of the ankle and knee. Participants with grade 2 of initial proprioception of the ankle and knee were classified into the normal group (n = 6). Participants with grade 0 or 1 of initial proprioception of the ankle and knee were classified into the abnormal group (n = 7).

In the normal group, the 10MWT, 6mWT, BBS, and WISCI-II were significantly improved (*p* = 0.028, 0.028, 0.028, and 0.027, respectively; Table 3); however, LEMS did not show a significant improvement (*p* = 0.068, Table 3). In the abnormal group, all outcome measures, including 10MWT, 6mWT, LEMS, proprioception of the ankle and knee, BBS, and WISCI-II, were significantly improved (*p* = 0.027, 0.028, 0.018, 0.027, 0.018, and 0.018, respectively; Table 3).

In the between-group comparisons, only the WISCI-II showed a statistically significant difference (*p* = 0.037, Table 4); a more significant improvement was observed in the normal group.

### 3.4. Correlation of Final Clinical Outcomes with Initial Outcome Measures

To reveal which initial outcome measures correlated with the final outcome results, we used the Spearman correlation. The final 10MWT was moderately correlated with the initial 10MWT (r = 0.568, *p* = 0.043) and strongly correlated with initial BBS (r = 0.739, *p* = 0.004) and WISCI-II (r = 0.882, *p* < 0.0001). The final 6mWT was strongly correlated with the initial BBS (r = 0.935, *p* < 0.0001) and WISCI-II (r = 0.705, *p* = 0.007). Final LEMS was moderately correlated with initial BBS (r = 0.616, *p* = 0.025) and strongly correlated with initial proprioception of the ankle and knee (r = 0.745, *p* = 0.003). Final proprioception of the ankle and knee was moderately correlated with initial LEMS (r = 0.683, *p* = 0.010) and initial WISCI-II (r = 0.611, *p* = 0.026). The final BBS score was strongly correlated with the initial BBS (r = 0.885, *p* < 0.0001) and WISCI-II (r = 0.751, *p* = 0.003). The final WISCI-II was moderately correlated with the initial 10MWT (r = 0.656, *p* = 0.015), 6mWT (r = 0.641, *p* = 0.018), proprioception of the ankle and knee (r = 0.582, *p* = 0.037), BBS (r = 0.589, *p* = 0.034), and WISCI-II (r = 0.679, *p* = 0.011) (Table 5).

### 3.5. Correlation between Final Clinical Outcomes

Finally, Spearman’s correlation was conducted again to inspect the correlation between the final clinical outcomes. The final 10MWT score was moderately correlated with the final WISCI-II (r = 0.578, *p* = 0.039) and strongly correlated with the final 6mWT (r = 0.830, *p* < 0.0001) and BBS (r = 0.803, *p* = 0.001). The final 6mWT was moderately correlated with final proprioception of the ankle and knee (r = 0.566, *p* = 0.044) and WISCI-II (r = 0.693, *p* = 0.009) and strongly correlated with the final BBS (r = 0.946, *p* < 0.0001). Final proprioception of the ankle and knee was moderately correlated with the final BBS (r = 0.609, *p* = 0.027). The final BBS score was strongly correlated with the final WISCI-II score (r = 0.718, *p* = 0.006) (Table 6).

## 4. Discussion

All clinical outcomes showed a significant improvement after 20 sessions of RAGT. End-effector RAGT improved gait speed (10MWT) and endurance (6mWT), LEMS, ankle and knee proprioception, balance ability (BBS), and ambulation capacity (WISCI-II). In previous studies on RAGT in SCI patients, the 6mWT, WISCI-II, and LEMS revealed improvements compared to the control group; however, the 10 MWT and BBS showed no significant improvement after the RAGT. [7,9,15,20,21,22] Most studies on RAGT in SCI patients used the 10MWT, 6mWT, LEMS, and WISCI-II as clinical outcomes. Only one study has used BBS as a clinical outcome [21], and no study has assessed proprioception in RAGT studies in SCI patients.

Before RAGT, two participants had WISCI-II level 8, and one participant had a WISCI-II level of 13. These three participants performed the 10MWT and 6mWT before RAGT. However, the others could not perform 10MWT and 6mWT before RAGT because their WISCI-II was less than 5. After 20 sessions of RAGT, 12 of the participants performed the 10MWT and 6mWT. The median values of improvement were 0.16 m/s and 52 m, respectively. Lam et al. [23] suggested 0.13 m/s for the 10MWT and 45.8 m for 6mWT as the clinically significant improvement. Therefore, our 10MWT and 6mWT improvements after RAGT were not only statistically but also clinically significant.

In previous studies that showed no statistically significant improvement in 10 MWT, the participants were subacute or chronic SCI patients [20,21,24,25]. However, in our study, most participants were in the acute phase. The median time from onset was only 48 days and ranged from 19 to 139 days. Therefore, we identified meaningful improvements in gait speed and endurance after RAGT.

To the best of our knowledge, this study is the first to use proprioception of the ankle and knee to evaluate end-effector RAGT effects in SCI patients. Only three articles have been published regarding end-effector RAGT in SCI patients [16,17,19]. One of them evaluated BBS for outcome measures, but this was a feasibility study in two patients with SCI [17].

Proprioception is an unconscious sensory perception of the body, joint position, and movement without visual feedback [1]. Despite having enough muscle strength, impaired proprioception leads to abnormal gait patterns.

In this study, we divided participants into normal and abnormal groups, followed by a subgroup analysis according to the initial proprioception of the ankle and knee. As a result of the between-group comparison, WISCI-II in the normal group showed statistically significant improvement compared to the abnormal group. This result means that patients with normal proprioception before the intervention will have a more favorable improvement in gait ability than those who had initial abnormal proprioception. This supports the relationship between proprioception and gait ability.

Postural control is also essential for the proprioception of gait [12]. Proprioception and postural control have relationships that affect each other. For postural control, complex interactions between the musculoskeletal and neural systems are required [12,26]. Therefore, impairment of the somatosensory system, including proprioception, leads to postural control disorders [12,27,28].

The end-effector robot produced a gait cycle according to the footplate trajectory. Unlike the exoskeletal-type robot, only the patient’s feet are attached to the robot’s footplate, and the knee and hip joints move freely. These free movements of the hip and knee joints provide “destabilization training.” In a previous study on end-effector RAGT in multiple sclerosis patients, postural stability (BBS) and balance confidence (Activities-Specific Balance Confidence Scale) improved as much as sensory integration balance training [11]. In addition, RAGT provides task-specific, repetitive training to patients, activates proprioceptors, and facilitates locomotor recovery [1].

Therefore, we hypothesized that the end-effector RAGT could improve proprioception and balance ability. As a result, proprioception of the ankle and knee showed statistically significant improvement after RAGT in this study. In addition, BBS was also significantly improved, unlike a previous study that conducted RAGT with exoskeletal type in SCI patients [21]. Thus, we concluded that end-effector RAGT could improve proprioception and balance ability by task-specific, repetitive destabilization training.

The other aim of this study was to determine which initial clinical outcome correlates with the final outcome and the correlations between final outcomes in patients with SCI. Initial BBS and WISCI-II scores were positively correlated with most of the final clinical outcome measures. The final BBS had a strong positive correlation with the final 10MWT, 6mWT, and WISCI-II. This means that postural control before intervention could play an essential role in the final favorable outcomes in gait. Postural control ability was positively correlated with gait ability.

Initial proprioception had a positive correlation with the final WISCI-II. This is the same result as the subgroup analysis according to initial proprioception. In addition, final proprioception had a moderate positive correlation with the 6mWT and BBS. This result supports the relationship between proprioception and gait ability.

The limitations of this study are the small sample size and the lack of a control group. A future randomized control study should be conducted to evaluate the effects of an end-effector RAGT on proprioception and balance ability in patients with SCI.

## 5. Conclusions

This study is the first to assess proprioception in order to evaluate the effect of an end-effector RAGT in patients with SCI. In addition, we evaluated BBS as an outcome measure that lacks evidence of the effect of an end-effector RAGT in patients with SCI. The results of this study suggest that the end-effector RAGT could act as task-specific, repetitive, and desensitization training to promote proprioception, balance ability, and walking ability. In addition, postural control ability and proprioception were positively correlated with gait ability.

## Figures and Tables

**Table 1:** Demographic and clinical characteristics (n = 13).

	Value
Age (yr.)	52 (19–85)
Sex	
Male	8
Female	5
Time from onset (day)	48 (19–139)
Etiology	
Traumatic	12
Non-traumatic	1
Level	
Tetraplegia	11
Paraplegia	2
AIS	
C	1
D	12

Values are presented as median (range) or number. AIS, American Spinal Injury Association impairment scale.

**Table 2:** Clinical outcome measures at baseline and after intervention.

	preRAGT	postRAGT	Effect Size	*p*-Value
10MWT (m/s)	0 (0–0)	0.16 (0.12–0.50)	1.20	0.002 **
6mWT (m)	0 (0–0)	52 (38–129)	1.03	0.002 **
LEMS	34 (30–37)	39 (38–40)	0.74	0.003 **
proprioception	10 (6–12)	12 (10–12)	0.63	0.027 *
BBS	14 (8–26)	38 (24–49)	1.48	0.001 **
WISCI-II	3 (0–4)	13 (9–19)	1.97	0.001 **

Values are presented as median (interquartile range). RAGT, robot-assisted gait training; 10MWT, 10 m walk test; 6mWT, 6 min walk test; LEMS, lower extremity motor scores; BBS, Berg Balance Scale; WISCI-II, Walking Index for Spinal Cord Injury-II. * Statistically significant at the Wilcoxon signed-rank test, *p* < 0.05. ** Statistically significant at the Wilcoxon singed-rank test, *p*-value < 0.01.

**Table 3:** Clinical outcome measures at baseline and after intervention by initial proprioception status.

		Normal Group (n = 6)				Abnormal Group (n = 7)		
	preRAGT	postRAGT	Effect Size	*p*-Value	preRAGT	postRAGT	Effect Size	*p*-Value
10MWT (m/s)	0 (0–0)	0.32 (0.13–0.48)	1.30	0.028 *	0 (0–0.07)	0.1 9(0.11–0.34)	0.91	0.027 *
6mWT (m)	0 (0–0)	92 (52–131)	1.49	0.028 *	0 (0–23)	40 (25–74)	0.51	0.028 *
LEMS	35 (33–39)	38 (37–40)	0.40	0.068	32 (29–36)	39 (38–40)	0.91	0.018 *
Proprioception	-	-		-	4 (4–6)	7 (6–7)	1.49	0.027 *
BBS	20 (12–28)	43 (31–49)	1.68	0.028 *	7 (5–16)	23 (18–45)	1.36	0.018 *
WISCI-II	3 (1–4)	15 (13–19)	3.17	0.027 *	2 (0–7)	11 (8–18)	1.35	0.018 *

Values are presented as median (interquartile range). RAGT, robot-assisted gait training; 10MWT, 10 m walk test; 6mWT, 6 min walk test; LEMS, lower extremity motor scores; BBS, Berg Balance Scale; WISCI-II, Walking Index for Spinal Cord Injury-II. * Statistically significant at the Wilcoxon signed-rank test, *p* < 0.05.

**Table 4:** Comparison of clinical outcomes by initial proprioception status.

	Normal Group (n = 6)	Abnormal Group (n = 7)	Effect Size	*p*-Value
Δ10MWT (m/s)	0.28 (0.13–0.48)	0.14 (0.11–0.32)	0.61	0.391
Δ6mWT (m)	91 (52–131)	26 (11–39)	1.28	0.063
ΔLEMS	2 (0–4)	4 (3–8)	0.81	0.097
ΔBBS	19 (17–23)	19 (11–23)	0.004	0.886
ΔWISCI-II	12 (10–13)	8 (7–10)	1.23	0.037 *

Values are presented as median (interquartile range). Δ, postRAGT-preRAGT; RAGT, robot-assisted gait training; 10MWT, 10 m walk test; 6mWT, 6 min walk test; LEMS, lower extremity motor scores; BBS, Berg Balance Scale; WISCI-II, Walking Index for Spinal Cord Injury-II. * Statistically significant at the Mann-Whitney U test, *p*-value < 0.05.

**Table 5:** Correlation final clinical outcomes with initial outcome measures.

			Initial Outcome Measures					
			10MWT	6mWT	LEMS	Proprioception	BBS	WISCI-II
Final outcome measures	10MWT	r	0.568 *	0.546	0.455	0.273	0.739 *	0.882 *
		*p*-value	0.043	0.053	0.118	0.366	0.004	<0.0001
	6mWT	r	0.446	0.379	0.215	0.448	0.935 **	0.705 **
		*p*-value	0.127	0.202	0.480	0.125	<0.0001	0.007
	LEMS	r	−0.138	−0.181	0.328	0.745 **	0.616 *	0.313
		*p*-value	0.653	0.555	0.273	0.003	0.025	0.297
	Proprio0ception	r	0.518	0.518	0.683 *	−0.036	0.069	0.611^*^
		*p*-value	0.070	0.070	0.010	0.908	0.823	0.026
	BBS	r	0.487	0.428	0.309	0.411	0.885 **	0.751 **
		*p*-value	0.091	0.145	0.304	0.164	<0.0001	0.003
	WISCI-II	r	0.656 *	0.641 *	0.390	0.582 *	0.589 *	0.679 *
		*p*-value	0.015	0.018	0.187	0.037	0.034	0.011

10 MWT, 10 m walk test; 6mWT, 6 min walk test; LEMS, lower extremity motor scores; BBS, Berg Balance Scale; WISCI-II, Walking Index for Spinal Cord Injury-II. * Statistically significant at the Spearman’s correlation, *p* < 0.05. ** Statistically significant at the Spearman correlation, *p*-value < 0.01.

**Table 6:** Correlation between final clinical outcomes.

		10MWT	6mWT	LEMS	Proprio-Ception	BBS	WISCI-II
10MWT	r		0.830 **	0.398	0.365	0.803 **	0.578 *
	*p*-value		<0.0001	0.178	0.221	0.001	0.039
6mWT	r	0.830 **		0.236	0.566 *	0.946 **	0.693 **
	*p*-value	<0.0001		0.438	0.044	<0.0001	0.009
LEMS	r	0.398	0.236		0.116	0.266	0.294
	*p*-value	0.178	0.438		0.707	0.381	0.330
Proprioception	r	0.365	0.566 *	0.116		0.609 *	0.475
	*p*-value	0.221	0.044	0.707		0.027	0.101
BBS	r	0.803 **	0.946 **	0.266	0.609^*^		0.718 **
	*p*-value	0.001	<0.0001	0.381	0.027		0.006
WISCI-II	r	0.578 *	0.693 **	0.294	0.475	0.718 **	
	*p*-value	0.039	0.009	0.330	0.101	0.006	

10MWT, 10 m walk test; 6mWT, 6 min walk test; LEMS, lower extremity motor scores; BBS, Berg Balance Scale; WISCI-II, Walking Index for Spinal Cord Injury-II. * Statistically significant at the Spearman’s correlation, *p* < 0.05. ** Statistically significant at the Spearman correlation, *p*-value < 0.01.

## Data Availability

The data presented in this study are available on request from the corresponding author. The data are not publicly available due to ethical and privacy restrictions.
